# Notice to Subscribers

**Published:** 1885-09

**Authors:** 


### NOTICE TO SUBSCRIBERS.

During September and October we will send out to subscribers a large number of bills. We hope that every one receiving a bill will promptly remit the amount. By doing this you will very materially aid us in giving you a first-class journal. Please do not put the matter off, but remit upon receipt of the bill.

### ITEMS.

THE British Medical Association has 11,249 members.

ACCORDING to Squibb's *Ephemeris*, it cost about six cents a grain to manufacture hydrochlorate of cocaine.

DR. FEHLING, of Stuttgart, the inventor of the well-known test for sugar, is dead. He died July 1st in his 73d year.

THREE deaths have been reported in Baltimore as having occurred from trichinosis following the eating of uncooked ham.

THE Mississippi Valley Medical Society will meet in Evansville, Indiana, September 8, 9 and 10, 1885. Gentlemen desiring to present papers, or wanting information concerning railroad rates or programme, will notify Dr. A. M. Owen, Evansville, Indiana.

DR. W. B. MATTHEWS, of Fort Valley, member of the Medical Association of Georgia since 1884, died in this city a few weeks since.

DR. D. B. SEARCY, one of the oldest members of the Medical Association of Georgia, died at his home near Bolingbroke, a few days since, at the age of 77 years. He was present and assisted in the organization of the Association in Macon in 1849. A more extended notice of him will appear in a future number of THE JOURNAL.

THE Illinois State Board of Health is now engaged in revising the "Official Register of Physicians and Midwives." Any notification of changes, omissions or errors will be regarded as a favor, as the Board wishes to make the coming register as correct as possible. Address Secretary State Board of Health, Springfield, Ill.

METEOROLOGICAL SUMMARY FOR THIS STATION FOR JULY, 1885.

| | |
|---|---|
| Highest Temperature, July 30th, | 91.2 |
| Lowest Temperature, July 2d, | 59.0 |
| Greatest Daily Range of Temperature, | 21.5 |
| Least Daily Range of Temperature, | 7.8 |
| Mean Daily Range of Temperature, | 15 4 |
| Mean Daily Dew-point, | 68.2 |
| Mean Daily Relative Humidity, | 73.7 |
| Prevailing Direction of Wind, | Southwest. |
| Total Movement of Wind, | 4,827 Miles. |
| Highest Velocity of Wind and Direction, | 24 Miles, W. and N. |
| Number of Foggy Days, | None. |
| Number of Clear Days, | 11 |
| Number of Fair Days, | 19 |
| Number of Cloudy Days, | 1 |
| Number of Days on which Rain or Snow fell, | 11 |

SALICYLIC ACID SUET IN HYPERIDROSIS.—Our Berlin correspondent writes : All the reports of the German army surgeons on experiments recently made with salicylic acid suet agree in recommending its use as a remedy for extreme sweating of the feet. It is composed of 2 parts of pure salicylic acid to

100 parts of best mutton suet. The War Minister has, therefore, permitted the introduction of this preparation into the army medical stores for the benefit of soldiers suffering from sweating feet or soreness from riding—*British Medical Journal.*

A NOVEL WAY OF REMOVING STONE FROM THE BLADDER.—A writer in the *British Medical Journal*, of August 8th, relates a case in which a patient removed forty-three calculi from his own bladder. It was accomplished by means of a large ear syringe, with a No. 10 catheter attached to it. While his bladder was full, he got on his knees, rolled the stone about till he considered he had it at the entrance to the urethra, then gently passed his catheter, with syringe attached, till he struck the stone; then, without displacing the stone, he gently withdrew the catheter about an inch and rapidly pulled out the piston, and, after some failures, succeeded in getting the stone into the urethra, when, by means of straining at first, and afterwards, when they came within reach of his fingers, by external manipulation, he removed them.

THE BRITISH MEDICAL ASSOCIATION AND HOMŒOPATHS.—At the recent meeting of the British Medical Association, the question of retaining homœopaths as members of the Association is reported on as follows : "The Council have had under their consideration the subject of admission and retention of homœopaths as members of the Association during the past year. An inquiry has been made throughout the thirty-three branches, and the result has been that there is evidence to the effect that a large majority of the members are adverse to the admission of homœopaths as members, but an equally large proportion are opposed to the idea of the expulsion of those members who have already gained admission into the ranks of the Association. Your Council, therefore, feel that this decided expression of opinion by the branches should guide the future action of the Association."

LAPAROTOMY FOR GUN-SHOT INJURY.—From the New York *Medical Journal* of August 8 we extract the following from a Washington letter: "Laparotomy with suture of the intestines was recently performed at the Providence Hospital by Dr. John B. Hamilton. The patient, a young mulatto, was shot in the abdomen three weeks ago. The wound was inflicted by a pistol carrying a 32 caliber ball. The missile severed a small artery in the mesentery, and made eleven wounds in the small intestines, and two in the ascending colon, and remained in the bowel. The operation was performed three hours after the accident. The artery was tied and the wounds were stitched with Lembert's suture. Fæces were passed by the natural channel on the seventh day, and on the twentieth day the patient was allowed to sit up, the abdominal wound having healed. The ball was passed with the fæces on the twelfth day.

THE INTERNATIONAL MEDICAL CONGRESS.—Rapid disintegration still characterizes the new organization of the Congress. This week we are called upon to chronicle more resignations, and the list includes one Vice-President of the Congress, three Vice-Presidents of Sections, and several members of Councils. The very large number of appointees who have declined to accept office under the New Orleans Committee are all Old Code men, both in principle and practice, and for the most part they are members of the American Medical Association. They recognize the falsity of the issues which were raised at New Orleans and they have promptly placed their emphatic seal of condemnation upon the most disgraceful piece of intrigue which has yet marred the history of that body, and which is in imminent danger of placing an ineffaceable stigma upon the good name of the whole American medical profession.

The clearest and cleanest way out of the false position into which the profession has been entrapped by the plotters at New

Orleans is for all appointees to discredit them completely by declining to accept office at their hands, and thus their organization must, of necessity, collapse. Already this has been largely done, and upwards of one hundred and twenty of the most eminent of their appointees have declined to be tools in their hands, and the sooner the remainder follow suit, the sooner will the way be opened to the profession to redeem its plighted honor.—*Medical News*.

The code question should really have nothing to do with an International Congress. Apart from this it is very well understood that the disturbance at New Orleans was incited by disappointed personal ambitions. The code and geographical distribution have been used to arouse opposition to the arrangements of the first committee.—*Boston Medical and Surgica Journal*.

Despite the very cheering assurances of the *Journal of the American Medical Association*, that the Ninth International Medical Congress will be conducted in the "most liberal and enlightened manner" by the "present able and judicious Committee of Arrangements," the progress of another week presents a wide-growing distrust of the recent work of the committee at Chicago. The withdrawal of the appointees of the committee in Philadelphia, Boston, Baltimore and Washington has been followed by similar resignations in Cincinnati, St. Louis, Chicago and in other localities. These declinations have come not only from the gentlemen originally appointed by the first committee, but, in a number of instances, the appointees of the present committee respectfully decline to hold the honors awarded them. Indeed, it seems to be quite apparent that the gifts of this committee will go begging unless some unseen power is raised up to prop its waning fortunes. Under existing circumstances it seems clearly the duty of this committee to abandon its work of reorganization as the most graceful and practical solution of the difficulties which embarrass it. By such a course the committee would in no sense lose the respect of the American profession. It has been placed in a false position by the American Medical Association. It has been called upon to perform a duty which no similar committee of the Association can perform under existing circumstances. The present status of the Congress is the result of a false and unnecessary issue, which can work no good to the American Medical Association. Its introduction into this discussion was totally unwarranted and unjustifiable. The "Code issue" is not an issue which should be raised in the organization of an International Medical Congress. At previous meetings of the Congress all questions of medical politics have been rigidly excluded, and such should have been the case in the organization of the present Congress.—*Maryland Medical Journal*, August 1, 1885.

Should the arrangements for the meeting be continued under the existing strained circumstances, nothing but disaster can ensue. It becomes an immediate question whether steps should not be taken for selecting another place than America as the scene of the next International Congress.—*The Medical Press and Circular*, July 22, 1885.

The Ninth International Medical Congress will be held according to appointment. The Association by which it was invited will see that it is officered in all its departments by as eminent and honorable men as exist in the medical profession of our country. And if the obstructionists desire to have any part in the good work, they will lose no time in converting their opposition into an honest co-operation in enabling the Committee of Arrangements, at its special meeting, on the 3d of September, to complete its work of organization in a satisfactory manner.—*Journal of the Am. Med. Association.*

It has been evident for sometime that the prospect for a successful International Congress in this country was very small. It is impossible to expect men of scientific attainments to cross the water to take part in a congress about which there is so much misunderstanding as in the present instance. It is exceedingly unpleasant to accept hospitalities in a house whose inmates are unable to agree as to the manner in which such hospitality shall be shown.—*Boston Med. and Surg. Journal*, August 13, 1885.

We consider it as now certain that the European members of the Congress have, through their Executive Committee, the power to prevent any material interference with the organization and work of the Congress itself, but while this does away with our fears, lest the progress and usefulness of these great international scientific gatherings should be checked by the action in this country, it increases our anxiety as to the effect of this discord upon our reputation abroad, and on our associations at home.

The action of the Original Committee—of the American Medical Association—and of the New Committee is now generally understood; and there does not seem to be much use in further comment and criticism upon what is past. The important question now is as to the future. Is there any way by which the impending disgrace can be averted? If there is, it must be such as will induce those who have withdrawn from the organization to return and co-operate heartily. To the best of our knowledge and belief, derived from an extensive correspondence and from personal interviews, there is but one way to do this, viz.: by dropping the Code question entirely, confirming all the appointments of the Original Committee, and leaving to the enlarged Committee which it created, including the Presidents of the Sections, the work of making additional appointments, completing the organization, and carrying out the work to its completion. If this be done, we believe that questions of appointments, etc., will be settled to general satisfaction, and that, although the difficulties of the work will be greatly increased, the Congress will be, what we all desire it should be, a great success.

If this be not done, we do not believe that the Congress will meet in this country in 1887.—*Medical News*, August 15, 1885.

